# Regular use of aspirin and other non-steroidal anti-inflammatory drugs and breast cancer risk for women at familial or genetic risk: a cohort study

**DOI:** 10.1186/s13058-019-1135-y

**Published:** 2019-04-18

**Authors:** Rebecca D. Kehm, John L. Hopper, Esther M. John, Kelly-Anne Phillips, Robert J. MacInnis, Gillian S. Dite, Roger L. Milne, Yuyan Liao, Nur Zeinomar, Julia A. Knight, Melissa C. Southey, Linda Vahdat, Naomi Kornhauser, Tessa Cigler, Wendy K. Chung, Graham G. Giles, Sue-Anne McLachlan, Michael L. Friedlander, Prue C. Weideman, Gord Glendon, Stephanie Nesci, kConFab Investigators, Irene L. Andrulis, Saundra S. Buys, Mary B. Daly, Mary Beth Terry

**Affiliations:** 1 Department of Epidemiology, Mailman School of Public Health, Columbia University, 722 W 168th St, New York, NY 10032 USA; 2 Centre for Epidemiology and Biostatistics, The University of Melbourne, Parkville, VIC 3010 Australia; 3 Department of Medicine and Stanford Cancer Institute, Stanford University School of Medicine, 780 Welch Road, Stanford, CA 94304 USA; 4 Department of Medical Oncology, Peter MacCallum Cancer Centre, 305 Grattan St, Melbourne, VIC 3000 Australia; 5 Sir Peter MacCallum Department of Oncology, The University of Melbourne, Parkville, VIC 3010 Australia; 6 Cancer Epidemiology, Cancer Council Victoria, 615 St Kilda Rd, Melbourne, VIC 3004 Australia; 7 Precision Medicine, School of Clinical Sciences at Monash Health, Monash University, Clayton, VIC 3168 Australia; 8 Lunenfeld-Tanenbaum Research Institute, Sinai Health System, 600 University Ave, Toronto, Ontario M5G 1X5 Canada; 9 Dalla Lana School of Public Health, University of Toronto, 155 College St, Toronto, Ontario M5T3M7 Canada; 10 Genetic Epidemiology Laboratory, Department of Pathology, The University of Melbourne, Parkville, VIC 3010 Australia; 11 Memorial Sloan Kettering Cancer Center, 300 East 66th Street, New York, NY 10065 USA; 12 C Anthony and Jean Whittingham Cancer Center, 34 Maple Street, Norwalk, CT 06856 USA; 13 Weill Cornell Medicine Breast Center, 428 E 72nd St, New York, NY 10021 USA; 14 Departments of Pediatrics and Medicine, Columbia University, 1150 St Nicholas Ave, New York, NY 10032 USA; 15 Herbert Irving Comprehensive Cancer Center, Columbia University Medical Center, 1130 St Nicholas Ave, New York, NY 10032 USA; 16 Department of Medicine, St Vincent’s Hospital, The University of Melbourne, Parkville, VIC 3010 Australia; 17 Department of Medical Oncology, St Vincent’s Hospital, 41 Victoria St, Fitzroy, VIC 3065 Australia; 18 Prince of Wales Clinical School, University of New South Wales, Sydney, NSW 2052 Australia; 19 Department of Medical Oncology, Prince of Wales Hospital, Barker St, Randwick, NSW 2031 Australia; 20 Division of Cancer Medicine, Peter MacCallum Cancer Centre, 305 Grattan St, Melbourne, VIC 3000 Australia; 21 Peter MacCallum Cancer Center, Melbourne, Victoria 3000 Australia; 22 Departments of Molecular Genetics and Laboratory Medicine and Pathobiology, University of Toronto, 164 College Street, Toronto, ON M5S 3G9 Canada; 23 Department of Medicine and Huntsman Cancer Institute, University of Utah Health, 2000 Cir of Hope Dr, Salt Lake City, UT 84103 USA; 24 Department of Clinical Genetics, Fox Chase Cancer Center, 333 Cottman Ave, Philadelphia, PA 19111 USA

**Keywords:** Breast cancer, Non-steroidal anti-inflammatory drugs, Family history, High-risk population

## Abstract

**Background:**

The use of aspirin and other non-steroidal anti-inflammatory drugs (NSAIDs) has been associated with reduced breast cancer risk, but it is not known if this association extends to women at familial or genetic risk. We examined the association between regular NSAID use and breast cancer risk using a large cohort of women selected for breast cancer family history, including 1054 *BRCA1* or *BRCA2* mutation carriers.

**Methods:**

We analyzed a prospective cohort (*N* = 5606) and a larger combined, retrospective and prospective, cohort (*N* = 8233) of women who were aged 18 to 79 years, enrolled before June 30, 2011, with follow-up questionnaire data on medication history. The prospective cohort was further restricted to women without breast cancer when medication history was asked by questionnaire. Women were recruited from seven study centers in the United States, Canada, and Australia. Associations were estimated using multivariable Cox proportional hazards regression models adjusted for demographics, lifestyle factors, family history, and other medication use. Women were classified as regular or non-regular users of aspirin, COX-2 inhibitors, ibuprofen and other NSAIDs, and acetaminophen (control) based on self-report at follow-up of ever using the medication for at least twice a week for ≥1 month prior to breast cancer diagnosis. The main outcome was incident invasive breast cancer, based on self- or relative-report (81% confirmed pathologically).

**Results:**

From fully adjusted analyses, regular aspirin use was associated with a 39% and 37% reduced risk of breast cancer in the prospective (HR = 0.61; 95% CI = 0.33–1.14) and combined cohorts (HR = 0.63; 95% CI = 0.57–0.71), respectively. Regular use of COX-2 inhibitors was associated with a 61% and 71% reduced risk of breast cancer (prospective HR = 0.39; 95% CI = 0.15–0.97; combined HR = 0.29; 95% CI = 0.23–0.38). Other NSAIDs and acetaminophen were not associated with breast cancer risk in either cohort. Associations were not modified by familial risk, and consistent patterns were found by *BRCA1* and *BRCA2* carrier status, estrogen receptor status, and attained age.

**Conclusion:**

Regular use of aspirin and COX-2 inhibitors might reduce breast cancer risk for women at familial or genetic risk.

**Electronic supplementary material:**

The online version of this article (10.1186/s13058-019-1135-y) contains supplementary material, which is available to authorized users.

## Background

Women vary greatly in their underlying familial risk of breast cancer (BC). Those with an affected first-degree relative are on average at 2-fold increased risk of BC. [1] Women with a *BRCA1* or *BRCA2* mutation are at about a 10-fold increased risk of BC, depending on their age, family history, and location of mutation [2]. The two leading risk-reduction strategies for women at increased BC risk are risk-reducing mastectomy, which could reduce risk by over 90% [3], and use of medications such as the selective estrogen receptor modulators or aromatase inhibitors, which reduce risk of estrogen receptor (ER)-positive BC by about 30–65% [4–6]. Despite the proven efficacy of these options, uptake remains low- and high-risk women often inquire about alternative BC prevention strategies [7–12]. Regular use of aspirin and other non-steroidal anti-inflammatory drugs (NSAIDs) including COX-2 inhibitors could be one such alternative. NSAIDs might impede tumor development and growth by modulating cellular proliferation and apoptosis, predominately by suppressing endogenous production of prostaglandin through the inhibition of cyclooxygenase (COX) enzyme activity, particularly COX-2, which is shown to be over-expressed in cancer cells [13, 14]. NSAIDs may also impede the development of ER positive BC through the inhibition of aromatase [13, 15]. The use of aspirin and other NSAIDs for BC prevention is an attractive strategy given that over-the-counter NSAIDs are inexpensive and widely available. However, even if regular NSAID use proves to be an effective BC prevention strategy, as with other risk-reducing options, the potential benefits of NSAIDs will need to be weighed against the potential harms of long-term use [16–21].

The cancer prevention effects of aspirin and other NSAIDs are well established for colon cancer [22, 23], and accumulating evidence from epidemiologic studies of women unselected for familial or genetic risk suggests that regular, long-term use of aspirin could reduce BC risk by about 14% [24, 25]. Comparable estimates have been reported for COX-2 inhibitors [26, 27]. However, the current body of evidence is far from conclusive [28], especially given that the only mature randomized controlled trial (RCT) of aspirin and primary prevention of BC did not find evidence for an effect, although no effect was found for colon cancer either [29]. While ongoing secondary prevention trials in women affected with breast cancer, such as the Aspirin for Breast Cancer (ABC) trial and Add-Aspirin trial [30, 31], will also inform this question, results from these trials have yet to be published. Recently published findings from the Aspirin in Reducing Events in the Elderly (ASPREE) found that cancer-related deaths, including BC, were higher in the aspirin group compared to those in the placebo group [21].

Little is known about whether aspirin and other NSAIDs reduce BC risk for women across the familial risk spectrum. For example, no study appears to have estimated the association for *BRCA1* and *BRCA2* mutation carriers. One study tested the association stratified by first-degree BC family history (12% of the overall sample) and found that regular aspirin use (≥ 6 times per week versus never) was associated with a reduced BC risk both for women with and without an affected first-degree relative (OR = 0.62, 95% CI = 0.41–0.93 and OR = 0.73, 95% CI = 0.61–0.88, respectively) [13]. The Sister Study, a prospective cohort study of women with a sister diagnosed with BC, also found a negative association between lifetime NSAID use (≥ 49 versus < 0.75 pill-years) and BC risk, although only for premenopausal women (HR = 0.66, 95% CI = 0.50–0.87; postmenopausal HR = 0.95, 95% CI = 0.82–1.09) [32]. However, both of these studies relied on a binary definition of family history, which discounts the fact that there is a strong gradient in risk due to underlying familial risk factors such as number of affected relatives and their age at diagnosis. Mathematical modeling demonstrates that in order to explain the average 2-fold increased risk of BC associated with having an affected first-degree relative, the risk of developing BC must vary by approximately 20-fold between people in the lowest quartile of familial risk versus the highest quartile of familial risk [33]. In our family cohort enriched with women with a family history of BC, remaining lifetime risk of BC ranges anywhere from < 1% to > 90% in women unaffected with BC at baseline [34]. It is possible to get a reliable estimate of this underlying familial risk, referred to as familial risk profile, from multi-generational breast and ovarian cancer history data using risk models such as the Breast Ovarian Analysis of Disease Incidence and Carrier Estimation Algorithm (BOADICEA), which includes consideration of *BRCA1* and *BRCA2* gene mutations [35–37]. In this study, we employed the BOADICEA model to evaluate associations of regular NSAID use and BC risk by familial risk profile using a large cohort of women enriched for family history, including 1054 women with a *BRCA1* or *BRCA2* mutation.

## Methods

### Study sample

This study was based on the Prospective Family Study Cohort (ProF-SC), which comprises baseline and follow-up data from the Breast Cancer Family Registry (BCFR) [38] and the Kathleen Cuningham Foundation Consortium for Research into Familial Breast Cancer (kConFab) [39]; additional details are available elsewhere [34]. These cohorts involved women affected with BC and included their affected and unaffected female relatives; ProF-SC is therefore enriched for familial risk of BC (82% with a first-degree relative and 95% with a first- or second-degree relative with BC). Baseline and follow-up questionnaires asked about personal and family history of BC, demographics, reproductive history, and lifestyle factors. Medication history was not asked about at baseline, but was included on follow-up questionnaires. Women were followed prospectively for cancer and other health outcomes for up to 20 years, and screening for germline *BRCA1* and *BRCA2* mutations has been conducted over time [39, 40]. The BCFR and kConFab received ethical approval by each participating study center’s institutional review board. All participants provided written informed consent.

### Prospective cohort

**Fig. 1:**
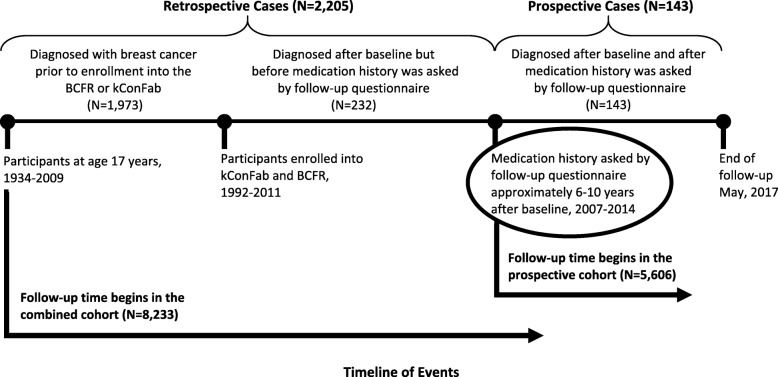
Overview of the timeline of events in the Prospective Family Study Cohort. Legend: BCFR, Breast Cancer Family Registry; kConFab, Kathleen Cuningham Foundation Consortium for Research into Familial Breast Cancer. The prospective cohort includes women who were enrolled before June 30, 2011, aged 18–79 years at follow-up, with data on regular NSAID use, and with no personal history of breast cancer when regular NSAID use was asked by follow-up questionnaire (*N* = 5606). The combined cohort includes all women enrolled before June 30, 2011, aged 18–79 years at baseline, with data on regular NSAID use asked by follow-up questionnaire. In both cohorts, women were censored at the earliest of the following events: risk-reducing bilateral mastectomy, age 80 years, loss to follow-up, or death

The prospective cohort included women who were enrolled in the BCFR or kConFab before June 30, 2011, aged 18–79 years at follow-up, who self-reported medication history by follow-up questionnaire, and had not undergone a bilateral mastectomy or been diagnosed with BC prior to follow-up questionnaire (*N* = 5606). To ensure that we did not include prevalent cancers in the prospective cohort, person-years were calculated from age at 2 months after the questionnaire with medication history was completed to age at first invasive breast cancer diagnosis, based on self- or relative-report and confirmed pathologically for 81% of cases, or censoring (Fig. 1). Women were censored at the earliest of the following events: risk-reducing bilateral mastectomy, age 80 years, loss to follow-up, or death.

### Combined cohort

The combined, retrospective and prospective, cohort included all women who were enrolled in the BCFR or kConFab before 30 June 2011, aged 18–79 years at baseline, who self-reported medication history by follow-up questionnaire. In addition to women in the prospective cohort, this included women who were diagnosed with BC prior to baseline (*N* = 1973), women who were diagnosed with BC after baseline but before follow-up questionnaire (*N* = 232), and women who were censored prior to follow-up questionnaire (*N* = 422). We excluded 16 cases missing BC diagnosis date, resulting in a final sample of 8233 women in the combined cohort. We used a general modeling approach for the combined cohort that was similar to the prospective cohort, except that we calculated person-years from age 17 years, 1 year prior to earliest age at diagnosis (Fig. 1).

### Exposure assessment

Medication history was asked by follow-up questionnaire including use of (1) aspirin, (2) COX-2 inhibitors, (3) ibuprofen and other non-selective NSAIDs, and (4) acetaminophen (paracetamol). Median time between baseline and follow-up questionnaire was 8.7 years. In the BCFR, women in the prospective cohort were asked if they had ever used each of the four types of medication for at least twice a week for 1 month or longer at any time in the past; kConFab participants were asked a slightly modified question that asked if medication use occurred for at least twice a week for more than 1 month. Participants were prompted with country-specific examples of each medication type to help with recall (e.g., Tylenol, Anacin-3, and Panadol were provided as examples of acetaminophen-based medications on the US-based questionnaire). Women in the combined cohort with a personal history of BC were asked if they had ever used (for at least twice a week for ≥ 1 month) the listed medications prior to diagnosis. Women who gave affirmative responses for a given medication were classified as regular users of that drug. Women who used these medications less frequently than twice per week for ≥ 1 month or never were classified as non-regular users. A subset of women (77% of the combined cohort) also reported total duration of regular medication use in either months or years.

### Statistical analysis

We used multivariable Cox proportional hazards regression, with age as the time scale, to estimate associations of regular medication use with BC risk. The proportionality assumption was assessed by evaluating Schoenfeld residuals. We estimated associations separately for regular use of aspirin, COX-2 inhibitors, ibuprofen and other NSAIDs, and acetaminophen, the latter as a negative control to determine if any associations observed between NSAIDs and BC risk reflected a non-specific use of analgesics [41]. We used a robust variance estimator to account for the family structure of the cohort. We stratified models by birth cohort (in 10-year categories) and adjusted for baseline age (continuous), race/ethnicity (non-Hispanic white versus otherwise), and study center (Model 1). We adjusted for familial risk profile using the 1-year BC risk score predicted from the BOADICEA model (Model 2) [35]. We also tested models adjusted for baseline health behaviors (never, former, current) including cigarette smoking, alcohol consumption, hormone therapy use, and hormonal birth control use, which collectively altered parameter estimates of some NSAID variables by > 10% (Model 3). Further adjustment for parity, breastfeeding, age at menarche, and body mass index did not alter the parameter estimates by > 10% and were not included in the final parsimonious model. Lastly, we tested a model further adjusted for use of the other three types of medications (Model 4). We estimated cross-product terms to test for multiplicative interactions between regular medication use and familial risk profile (continuous). We also plotted the predicted age-specific absolute cumulative risk for women with different familial risk based on BOADICEA and underlying age-specific incidences from the Surveillance, Epidemiology, and End Results Program [42–44]. We chose three scenarios of familial risk: 12% (population average), 20–30% (moderate familial risk), and > 30% (high familial risk) full lifetime BC risk, and two scenarios of medication use: regular aspirin user and non-regular aspirin user.

### Subgroup analyses

Using the combined cohort, we estimated associations stratified by gene mutation carrier status defined as non-carriers (either true negative or not tested), *BRCA1* carriers, or *BRCA2* carriers. We conducted a sensitivity analysis using the weighting approach of Antoniou et al. (2005) [45] to account for non-random selection of mutation carriers. Weighting did not substantively alter medication-associated risk estimates or their standard errors. We also estimated associations by tumor ER status (positive or negative); the alternative ER subtype was censored at diagnosis. For example, ER-negative BC cases were censored at age at diagnosis in the analysis of ER-positive BC. Finally, we fitted attained age models, truncating follow-up time at ages 45, 55, and 65 years, to assess associations for younger women.

### Sensitivity analyses

Although our primary analysis was based on regular NSAID use, we did an additional analysis examining duration of use (categorized as ≥ 5 years versus < 5 years) for women who provided this information. We conducted a sensitivity analysis excluding women who reported tamoxifen use at baseline (*N* = 64) and found this did not alter estimates. We conducted another sensitivity analysis further adjusting for diabetes and other cancers to account for comorbidities and found that this also did not appreciably alter estimates. To account for missing data, we conducted a sensitivity analysis using multiple imputation by chained equations, which produced comparable findings (data not shown). Statistical significance was determined as *p* < 0.05 for a two-sided hypothesis test. Analyses were conducted using Stata 15.1 (College Station, TX) [46].

## Results

There were 139 incident cases of BC in the prospective cohort over 27,923 person-years. Prevalences of medication use were similar in the prospective and combined cohorts (18% and 19% for aspirin, 9% and 8% for COX-2 inhibitors, 17% and 19% for ibuprofen, and 17% and 17% for acetaminophen, respectively). Tetrachoric correlations between medications were statistically significant, but all ≤ 0.47 (see Additional File 1). At study enrollment, regular aspirin users were older on average than non-regular users (53.1 versus 41.7 years). Regular aspirin users were also less likely to smoke cigarettes, consume alcohol, or use hormonal birth control than non-regular users, but were more likely to use hormone therapy and other types of medication (Table 1). On average, regular aspirin users had a higher 1-year BOADICEA risk score than non-regular users, which is influenced by baseline age; a smaller proportion of aspirin users had a known mutation in the *BRCA1* or *BRCA2* gene. The distribution of 1-year BOADICEA risk score by regular medication use is provided in the supplemental materials (see Additional File 2).

**Table 1:** Baseline characteristics of women in the Prospective Family Study Cohort by regular aspirin use

	Prospective cohort^a^		Combined cohort^b^	
	Non-regular user	Regular user	Non-regular User *N* = 6636	Regular user
	*N* = 4616	*N* = 990		*N* = 1597
Baseline characteristic	Mean (SD)	Mean (SD)	Mean (SD)	Mean (SD)
Age, years	41.7 (12.7)	53.1 (11.7)	44.0 (12.9)	54.2 (11.5)
Race and ethnicity, No. (%)				
Non-Hispanic white	4269 (92.5)	888 (89.7)	5860 (88.3)	1366 (85.5)
Other	274 (5.9)	94 (9.5)	694 (10.5)	223 (14.0)
Missing	73 (1.6)	8 (0.8)	82 (1.2)	8 (0.5)
Cigarette smoking, No. (%)				
Never	2688 (58.2)	575 (58.1)	3862 (58.2)	896 (56.1)
Former	1088 (23.6)	267 (27.0)	1705 (25.7)	492 (30.8)
Current	469 (10.2)	91 (9.2)	663 (10.0)	140 (8.8)
Missing	371 (8.0)	57 (5.8)	406 (6.1)	69 (4.3)
Alcohol consumption, No. (%)				
Never	1852 (40.1)	532 (53.7)	2943 (44.4)	841 (52.7)
Former	755 (16.4)	129 (13.0)	1062 (16.0)	220 (13.8)
Current	1924 (41.7)	318 (32.1)	2514 (37.9)	513 (32.1)
Missing	85 (1.8)	11 (1.1)	117 (1.8)	23 (1.4)
Hormone therapy use, No. (%)				
Never	3717 (80.5)	542 (54.8)	5255 (79.2)	895 (56.0)
Former	409 (8.9)	191 (19.3)	758 (11.4)	361 (22.6)
Current	430 (9.3)	230 (23.2)	522 (7.9)	290 (18.2)
Missing	60 (1.3)	27 (2.7)	101 (1.5)	51 (3.2)
Hormonal birth control use, No. (%)				
Never	635 (13.8)	253 (25.6)	1224 (18.4)	480 (30.1)
Former	3043 (65.9)	659 (66.6)	4373 (65.9)	1015 (63.6)
Current	900 (19.5)	59 (6.0)	973 (14.7)	69 (4.3)
Missing	38 (0.8)	19 (1.9)	66 (1.0)	33 (2.1)
COX-2 inhibitors, No. (%)				
Non-regular user	4227 (91.6)	814 (82.2)	6140 (92.5)	1366 (85.5)
Regular user	358 (7.8)	161 (16.3)	428 (6.5)	199 (12.5)
Missing	31 (0.7)	15 (1.5)	68 (1.0)	32 (2.0)
Ibuprofen and other NSAIDs^c^, No. (%)				
Non-regular user	3911 (84.7)	714 (72.1)	5512 (83.1)	1139 (71.3)
Regular user	682 (14.8)	258 (26.1)	1063 (16.0)	426 (26.7)
Missing	23 (0.5)	18 (1.8)	61 (0.9)	32 (2.0)
Acetaminophen, No. (%)				
Non-regular user	3859 (83.6)	740 (74.8)	5597 (84.3)	1214 (76.0)
Regular user	737 (16.0)	238 (24.0)	985 (14.8)	361 (22.6)
Missing	20 (0.4)	12 (1.2)	54 (0.8)	22 (1.4)
BOADICEA 1-year risk score, %	0.51 (0.71)	0.68 (0.85)	0.50 (0.72)	0.64 (0.83)
Mutation carrier status, No. (%)				
Non-carrier^d^	4070 (88.2)	915 (92.4)	5739 (86.5)	1440 (90.2)
*BRCA1* mutation carrier	292 (6.3)	37 (3.7)	500 (7.5)	84 (5.3)
*BRCA2* mutation carrier	254 (5.5)	38 (3.8)	397 (6.0)	73 (4.6)

^a^Includes women with no personal history of breast cancer when regular medication use was asked by questionnaire (*N* = 5606)

^b^Includes retrospective breast cancer cases (diagnosed prior to baseline and/or follow-up questionnaire) and prospective breast cancer cases (diagnosed after follow-up questionnaire) (*N* = 8233)

^c^Non-steroidal anti-inflammatory drugs

^d^Includes true negatives and women who did not undergo genetic testing for *BRCA1* and *BRCA2*

As shown in Table 2, from analysis of the prospective cohort, regular aspirin use was associated with a 39% reduced BC risk in the fully adjusted model (Model 4: prospective hazard ratio (HR_p_) = 0.61, 95% confidence interval (CI) = 0.33 to 1.14); a very similar, but more precise, estimate was obtained from analysis of the combined cohort (Model 4: combined hazard ratio (HR_c_) = 0.63, 95% CI = 0.57 to 0.71). When we considered duration of aspirin use in the combined cohort, < 5 years versus never use was associated with an estimated 32% reduced BC risk (HR_c_ = 0.68, 95% CI = 0.58 to 0.80); ≥ 5 years versus never use was associated with an estimated 66% reduced BC risk (HR_c_ = 0.34, 95% CI = 0.27 to 0.44). From fully adjusted models, regular use of COX-2 inhibitors was associated with a 61% reduced BC risk for the prospective cohort (Model 4: HR_p_ = 0.39, 95% CI = 0.15 to 0.97) and a 71% reduced BC risk for the combined cohort (Model 4: HR_c_ = 0.29, 95% CI = 0.23 to 0.38). After adjusting for regular use of other medications (Model 4), regular use of ibuprofen and other NSAIDs (or ibuprofen exclusively) was not associated with BC risk, nor was acetaminophen.

**Table 2:** Adjusted hazard ratios (HRs) and 95% confidence intervals (CI) of breast cancer risk comparing regular medication users with non-regular users in the Prospective Family Study Cohort

	Number of events	Person-years	Model 1^a^	Model 2^b^	Model 3^c^	Model 4^d^
Medication			HR (95% CI)	HR (95% CI)	HR (95% CI)	HR (95% CI)
Aspirin-based medications						
*Prospective cohort*^*e*^						
Non-regular user	124	23,545	1.00 (ref)	1.00 (ref)	1.00 (ref)	1.00 (ref)
Regular user	15	4378	0.57 (0.32, 1.00)	0.56 (0.32, 1.00)	0.55 (0.29, 1.06)	0.61 (0.33, 1.14)
*Combined cohort*^*f*^						
Non-regular user	1838	236,452	1.00 (ref)	1.00 (ref)	1.00 (ref)	1.00 (ref)
Regular user	503	71,610	0.57 (0.51, 0.63)	0.57 (0.51, 0.63)	0.60 (0.54, 0.67)	0.63 (0.57, 0.71)
COX-2 inhibitors						
*Prospective cohort*^*e*^						
Non-regular user	133	25,203	1.00 (ref)	1.00 (ref)	1.00 (ref)	1.00 (ref)
Regular user	6	2519	0.39 (0.17, 0.90)	0.38 (0.16, 0.88)	0.37 (0.14, 0.94)	0.39 (0.15, 0.97)
*Combined cohort*^*f*^						
Non-regular user	2236	274,964	1.00 (ref)	1.00 (ref)	1.00 (ref)	1.00 (ref)
Regular user	66	29,076	0.25 (0.20, 0.32)	0.26 (0.20, 0.33)	0.28 (0.22, 0.36)	0.29 (0.23, 0.38)
Ibuprofen and other NSAIDs^g^						
*Prospective cohort*^*e*^						
Non-regular user	116	23,081	1.00 (ref)	1.00 (ref)	1.00 (ref)	1.00 (ref)
Regular user	22	4645	0.93 (0.57, 1.52)	0.94 (0.57, 1.54)	1.04 (0.61, 1.76)	1.24 (0.72, 2.12)
*Combined cohort*^*f*^						
Non-regular user	1836	248,675	1.00 (ref)	1.00 (ref)	1.00 (ref)	1.00 (ref)
Regular user	459	55,695	0.79 (0.71, 0.88)	0.80 (0.72, 0.89)	0.84 (0.76, 0.94)	0.93 (0.83, 1.04)
Ibuprofen-based medications^h^						
*Prospective cohort*^*e*^						
Non-regular user	39	8170	1.00 (ref)	1.00 (ref)	1.00 (ref)	1.00 (ref)
Regular user	11	2888	0.68 (0.34, 1.37)	0.69 (0.35, 1.39)	0.84 (0.42, 1.69)	0.99 (0.49, 2.01)
*Combined cohort*^*f*^						
Non-regular user	1685	134,961	1.00 (ref)	1.00 (ref)	1.00 (ref)	1.00 (ref)
Regular user	439	42,044	0.77 (0.61, 0.96)	0.74 (0.59, 0.94)	0.81 (0.64, 1.02)	0.83 (0.65, 1.05)
Acetaminophen-based medications						
*Prospective cohort*^*e*^						
Non-regular user	116	23,415	1.00 (ref)	1.00 (ref)	1.00 (ref)	1.00 (ref)
Regular user	23	4370	0.96 (0.61, 1.53)	1.00 (0.63, 1.60)	0.86 (0.49, 1.49)	0.96 (0.55, 1.65)
*Combined cohort*^*f*^						
Non-regular user	1985	250,745	1.00 (ref)	1.00 (ref)	1.00 (ref)	1.00 (ref)
Regular user	318	54,649	0.82 (0.73, 0.93)	0.82 (0.73, 0.93)	0.83 (0.73, 0.95)	0.98 (0.85, 1.12)

^a^Model 1 is adjusted for race/ethnicity, study center, and baseline age; stratified by birth cohort

^b^Model 2 is further adjusted for familial risk profile

^c^Model 3 is further adjusted for cigarette smoking, alcohol consumption, hormone therapy use, and hormonal birth control use

^d^Model 4 is further adjusted for regular use of the other types of medication. For example, the Model 4 estimates for regular aspirin use are adjusted for regular use of COX-2 inhibitors, ibuprofen and other NSAIDs, and acetaminophen

^e^Includes women with no personal history of breast cancer when regular medication use was asked by questionnaire (*N* = 5606)

^f^Includes retrospective breast cancer cases (diagnosed prior to baseline and/or follow-up questionnaire) and prospective breast cancer cases (diagnosed after follow-up questionnaire) (*N* = 8233)

^g^Non-steroidal anti-inflammatory drugs

^h^kConFab participants are excluded from these models because they were only asked about regular use of other NSAIDs, including ibuprofen, but not about ibuprofen specifically

**Fig. 2:**
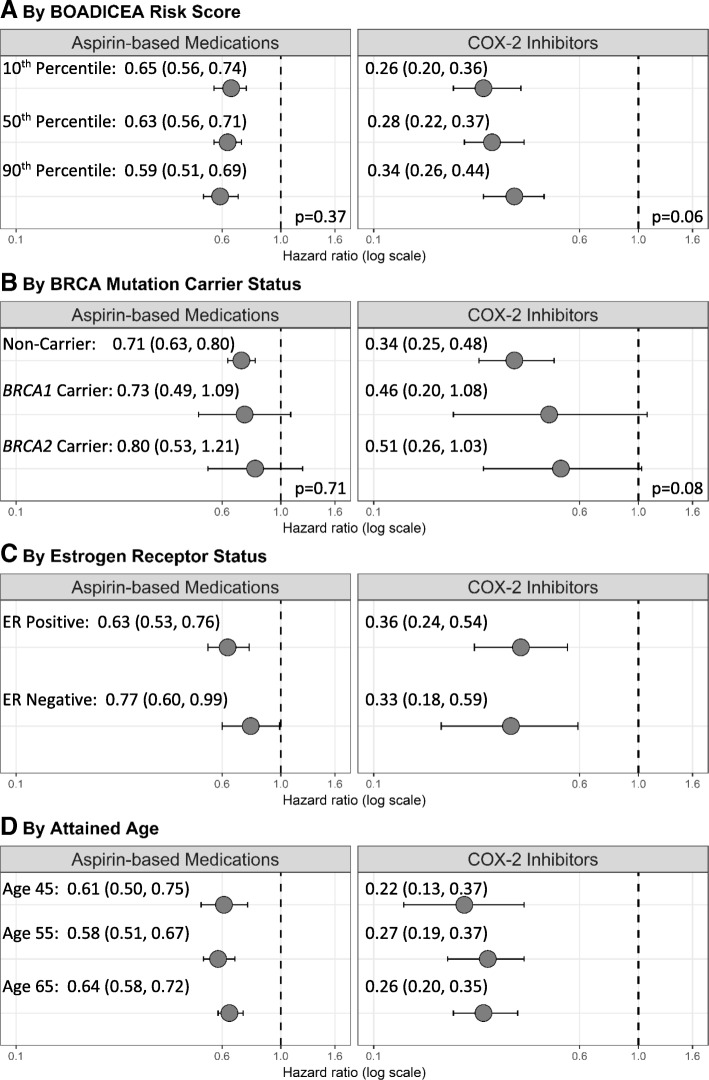
Adjusted hazard ratios and 95% confidence intervals of breast cancer risk comparing regular users of aspirin and COX-2 inhibitors with non-regular users by subgroups from analysis of the combined cohort of the Prospective Family Study Cohort (*N* = 8233). Legend: Models are adjusted for race/ethnicity, study center, baseline age, familial risk profile, cigarette smoking, alcohol consumption, hormone therapy use, hormonal birth control use, and regular use of other medications; stratified by birth cohort. Sample sizes: non-carriers (includes true negatives and women who did not undergo genetic testing) *N* = 6395; *BRCA1* mutation carriers *N* = 506; *BRCA2* mutation carriers *N* = 418; ER status: *N* = 7319; attained age 45: *N* = 2222; attained age 55: *N* = 4401; attained age 65: *N* = 6325. Alternative ER subtypes were censored at diagnosis (e.g., ER negative and ER status missing breast cancers censored at age at diagnosis in the analysis of ER positive breast cancer). *P* values are for the Wald chi-square test statistic for the interaction between categories of familial risk profile or *BRCA* carrier status and regular medication use

Associations of regular use of aspirin and Cox-2 inhibitors with reduced breast cancer risk were not modified by familial risk profile as estimated by the BOADICEA 1-year BC risk score (Fig. 2a). When we stratified the combined cohort by gene mutation carrier status (Fig. 2b), similar HR estimates were found for women not known to be mutation carriers (HR_c_ = 0.71, 95% CI = 0.63 to 0.80), *BRCA1* carriers (HR_c_ = 0.73, 95% CI = 0.49 to 1.09), and *BRCA2* carriers (HR_c_ = 0.80, 95% CI = 0.53 to 1.21), although the latter two confidence intervals were wide. Consistent estimates were also found for the association between COX-2 inhibitors and BC risk for women not known to be mutation carriers (HR_c_ = 0.34, 95% CI = 0.25 to 0.48), *BRCA1* carriers (HR_c_ = 0.46, 95% CI = 0.20 to 1.08), and *BRCA2* carriers (HR_c_ = 0.51, 95% CI = 0.26 to 1.03); confidence intervals were again wide for known mutation carriers. Similar associations were also found when we stratified by ER status (Fig. 2c) and when we assessed attained age models (Fig. 2d).

**Fig. 3:**
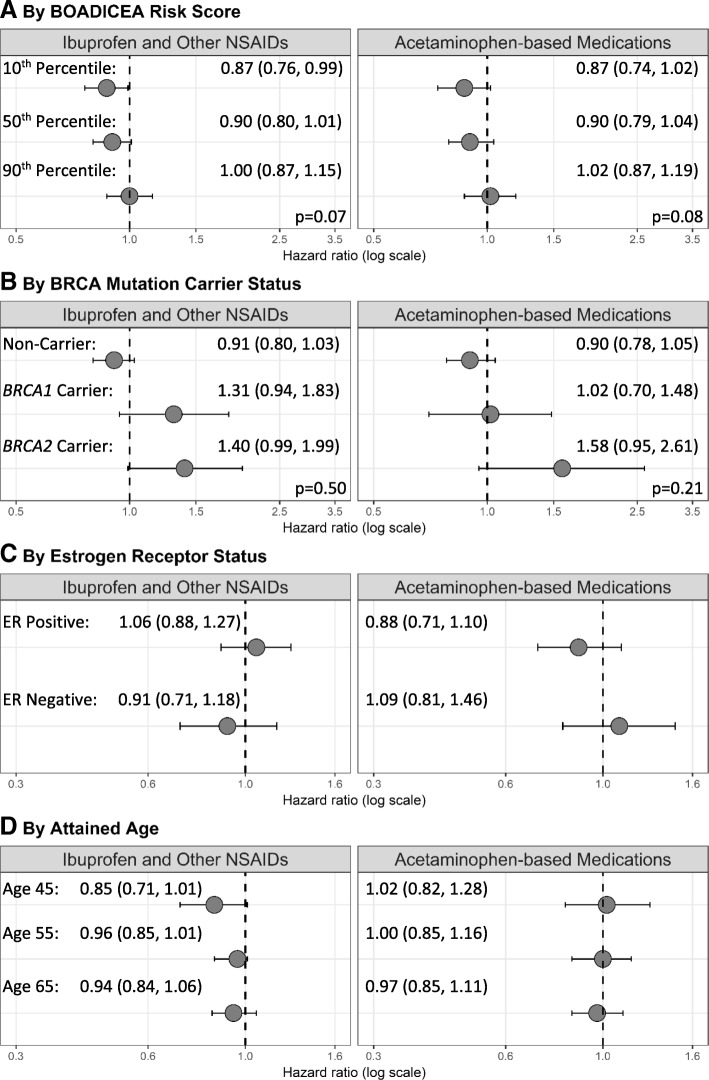
Adjusted hazard ratios and 95% confidence intervals of breast cancer risk comparing regular users of ibuprofen and other NSAIDs and acetaminophen with non-regular users by subgroup in the combined cohort of the Breast Cancer Prospective Family Study Cohort (*N* = 8233). Legend: Models are adjusted for race/ethnicity, study center, baseline age, familial risk profile, cigarette smoking, alcohol consumption, hormone therapy use, hormonal birth control use, and regular use of other medications; stratified by birth cohort. Sample sizes: non-carriers (includes true negatives and women who did not undergo genetic testing) *N* = 6395; *BRCA1* mutation carriers *N* = 506; *BRCA2* mutation carriers *N* = 418; ER status: *N* = 7319; attained age 45: *N* = 2222; attained age 55: *N* = 4401; attained age 65: *N* = 6325. Alternative ER subtypes were censored at diagnosis (e.g., ER negative and ER status missing breast cancers censored at age at diagnosis in the analysis of ER-positive breast cancer). *P* values are for the Wald chi-square test statistic for the interaction between categories of familial risk profile or *BRCA* carrier status and regular medication use

No associations with BC risk were found for regular use of ibuprofen and other NSAIDs or acetaminophen from subgroup analyses by familial risk profile, *BRCA1* or *BRCA2* mutation carrier status, tumor ER status, or attained age (Fig. 3).

**Fig. 4:**
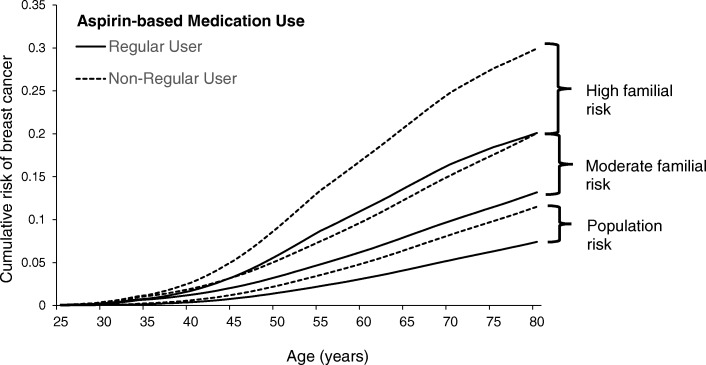
Cumulative risk of breast cancer by regular aspirin use and underlying familial risk. Legend: Predicted age-specific cumulative risk (from birth) for breast cancer, by regular aspirin use and familial risk, where 12% lifetime risk is approximately the population risk of breast cancer by age 80 years, where moderate familial risk (> 20–30% full lifetime BOADICEA) is equivalent to having one affected first-degree relative, and high familial risk (> 30% full lifetime BOADICEA) is equivalent to having two affected first-degree relatives

Figure 4 shows the overall implications of the study estimates on the predicted age-specific BC cumulative risk for non-regular users of aspirin and regular users of aspirin with different familial risk profiles. In terms of absolute risk, the risk difference between regular users and non-regular users is greater for women with higher familial risk. For cumulative BC risk to age 80 years, the risk difference is 4.1%, 6.9%, and 9.8% for women at population average risk, moderate familial risk, and high familial risk, respectively.

## Discussion

From studying a prospective and combined (prospective and retrospective) cohort enriched with women having a family history of BC across a wide range of absolute predicted familial BC risk (10-year risk: mean, 5.3%; range, < 0.1–68.5%), we found regular aspirin use to be associated with a 39% and 37% reduction in BC risk in the two cohorts, respectively. Regular use of COX-2 inhibitors was associated with a 61% and 71% reduction in BC risk in the two cohorts, respectively. The strength of these associations did not differ by familial risk or mutation status, and although not nominally significant, negative associations were found for both *BRCA1* and *BRCA2* mutation carriers. Negative associations were also found for younger women based on attained age models examining risk up to age 45 years.

Our findings are consistent with most, but not all, other studies of NSAIDS and BC risk conducted using samples of average-risk women unselected for family history [24]. As previously noted, the only RCT of aspirin and BC risk as the primary endpoint did not observe an association after 10 years of follow-up (relative risk = 0.98; 95% CI = 0.89–1.08), but this could reflect the fact that participants were randomized to a low dose of aspirin (100 mg every other day) [29]. We found that regular use of aspirin and COX-2 inhibitors was associated with reduced risk of both ER-positive and ER-negative BCs, which again aligns with most, but not all [41, 47, 48], other studies that considered hormone receptor status [49]. This suggests that these NSAIDs might operate through multiple underlying biological pathways to lower BC risk, including a direct effect through ER mediated signaling pathways [13, 41], the COX-2 pathway [13, 50], phosphatidylinositol 3-kinase down-regulation [13, 51], B cell lymphoma 2-mediated apoptosis [13, 52], or epidermal growth factor receptor inhibition and p53 acetylation [13, 53].

We no longer found a statistically significant association between ibuprofen and other NSAIDs and BC risk after we adjusted for regular use of other medications. This could be because ibuprofen does not have an effect on BC risk, as some NSAIDs might inhibit COX-2 more intensely than others [26]. It could also reflect differences in the duration and frequency of ibuprofen and other NSAID use compared with aspirin and COX-2 inhibitors. Women might be more likely to use aspirin regularly because of its anticlotting effect [27, 54, 55], which ibuprofen does not deliver [56]. We also found no evidence of an association between acetaminophen, an analgesic with minimal anti-inflammatory action, and BC risk. This supports the known pharmacological effects of NSAIDs and minimizes concerns that the associations we found are due to confounding from other unmeasured lifestyle factors associated with regular analgesic use [41, 57].

The present study has several strengths. Most notably, we used data from a large cohort of women that is enriched for familial or genetic BC risk. This allowed us to test if associations between NSAIDs and BC risk vary in strength across a wide range of familial BC risk. Another strength is the use of the BOADICEA [34] to estimate a woman’s familial risk profile [35]. We also estimated associations by known *BRCA1* and *BRCA2* mutation carrier status. Although we had low statistical power, this is the first study to consider carrier-specific associations between NSAIDs and BC risk. One limitation of the present study is the use of binary measures of regular medication use. We also did not have information on dosage, and we had only limited data to examine duration of use. We recognize that these factors need to be considered to fully understand the association of NSAIDs with BC risk. Another limitation is that our exposure measures were retrospective, and thus, recall bias is a concern when interpreting the estimates from the combined cohort analyses. Survival bias is another potential limitation, but the consistency of associations between the prospective and combined cohorts supports that biases that operate differently in prospective and retrospective settings are unlikely to explain these findings. Confounding by indication could also be of concern, given that we did not have information on the reason for medication use. However, attained age models estimated similar associations in young women for whom comorbidities are unlikely, and the sensitivity analysis that further adjusted for diabetes and other cancers produced comparable estimates.

## Conclusion

In summary, our findings add to growing evidence for an association between regular use of aspirin and COX-2 inhibitors and reduced BC risk. The potential impact of using these medications for primary BC prevention is underscored by the fact that associations were not modified by familial risk, and suggestive negative associations were found for *BRCA1* and *BRCA2* mutation carriers. This means that individuals at higher risk of BC could benefit even more in terms of absolute risk reduction from modifying medication use. Additionally, our findings suggest that regular use of aspirin and COX-2 inhibitors are associated with BC risk independent of ER status, which is important because risk-reducing medications are currently only available for ER-positive BC. Although RCTs are ultimately needed to confirm associations of NSAIDs with BC risk, our findings support that the consistent results seen in observational studies of average-risk women may extend to women at the higher range of absolute BC risk.

## Additional files

Additional file 1: Correlation between regular use of medications in the combined cohort of the Prospective Family Study Cohort (*N* = 8233). Additional File 1 presents tetrachoric correlations and odds ratios comparing regular use of each of the four medications (aspirin, Cox-2 inhibitors, ibuprofen, and acetaminophen) that were included in the analysis. (DOCX 15 kb)

Additional file 2: Distribution of BOADICEA 1-year risk scores by medication use in the combined cohort of the Prospective Family Study Cohort (*N* = 8233). Additional File 2 presents overlapping histograms of the distribution of BOADICEA one-year risk score by medication use (regular users versus non-regular users). (DOCX 45 kb)

## Abbreviations

ABC: Aspirin for Breast Cancer
ASPREE: Aspirin in Reducing Events in the Elderly
BC: Breast cancer
BCFR: Breast Cancer Family Registry
BOADICEA: Breast Ovarian Analysis of Disease Incidence and Carrier Estimation Algorithm
CI: Confidence interval
COX: Cyclooxygenase
ER: Estrogen receptor
HR: Hazard ratio
kConFab: Kathleen Cuningham Foundation Consortium for Research into Familial Breast Cancer
NSAID: Non-steroidal anti-inflammatory drug
OR: Odds ratio
ProF-SC: Prospective Family Study Cohort
RCT: Randomized controlled trial
