# Supplementary material for: Regular use of aspirin and other non-steroidal anti-inflammatory drugs and breast cancer risk for women at familial or genetic risk: a cohort study
Source: Breast Cancer Res. 2019 Apr 18;21:52. doi: 10.1186/s13058-019-1135-y (PMC6471793; doi:10.1186/s13058-019-1135-y)
Supplement: Supplementary file 1 — Correlation between regular use of medications in the combined cohort of the Prospective Family Study Cohort (N = 8233). Additional File 1 presents tetrachoric correlations and odds ratios comparing regular use of each of the four medications (aspirin, Cox-2 inhibitors, ibuprofen, and acetaminophen) that were included in the analysis. (DOCX 15 kb) [file 13058_2019_1135_MOESM1_ESM.docx]

**Additoinal File 1. Correlation between regular use of medications in the the combined cohort of the Prospective Family Study Cohort (N=8,233)**

|  | **Aspirin** | **Cox-2 Inhibitors** | **Ibuprofen** | **Acetaminophen** |
| --- | --- | --- | --- | --- |
| **Aspirin** | 1.00 | 2.09 (1.74, 2.50) | 1.94 (1.70, 2.21) | 1.69 (1.48,1.94) |
| **Cox-2 Inhibitors** | 0.22* | 1.00 | 3.17 (2.67, 3.77) | 4.35 (3.66, 5.17) |
| **Ibuprofen** | 0.22* | 0.34* | 1.00 | 4.48 (3.93, 5.10) |
| **Acetaminophen** | 0.17* | 0.42* | 0.47* | 1.00 |

Notes: Tetrachoric correlations are presented in white cells; p-values <0.05 indicated with an asterisk (*). Odds ratios predicted from bivariate logistic regression models are presented in gray cells.
