# Supplementary material for: Regular use of aspirin and other non-steroidal anti-inflammatory drugs and breast cancer risk for women at familial or genetic risk: a cohort study
Source: Breast Cancer Res. 2019 Apr 18;21:52. doi: 10.1186/s13058-019-1135-y (PMC6471793; doi:10.1186/s13058-019-1135-y)
Supplement: Supplementary file 2 — Distribution of BOADICEA 1-year risk scores by medication use in the combined cohort of the Prospective Family Study Cohort (N = 8233). Additional File 2 presents overlapping histograms of the distribution of BOADICEA one-year risk score by medication use (regular users versus non-regular users). (DOCX 45 kb) [file 13058_2019_1135_MOESM2_ESM.docx]

**Additoinal File 2. Distribution of BOADICEA one-year risk scores by medication use in the combined cohort of the Prospective Family Study Cohort (N=8,233)**

**A. Aspirin-based Medications B. Cox-2 Inhibitors**

**C. Ibuprofen and Other NSAIDs D. Acetaminophen-based Medications**
